# Using REACH for the EU Environmental Footprint: Building a Usable Ecotoxicity Database, Part I

**DOI:** 10.1002/ieam.4168

**Published:** 2019-08-14

**Authors:** Erwan Saouter, Fabrizio Biganzoli, Rana Pant, Serenella Sala, Donald Versteeg

**Affiliations:** ^1^ European Commission, Joint Research Centre (JRC) Ispra Italy; ^2^ EcoStewardship, Cincinnati Ohio USA

**Keywords:** REACH, Ecotoxicity, Environmental Footprint, Hazard values, LCA

## Abstract

The European Union Environmental Footprint (EU‐EF) is a harmonized method to measure and communicate the life cycle environmental performance of products and organizations. Among 16 different impact categories included in the EU‐EF, 1 focuses on the impact of substances on freshwater ecosystems and requires the use of toxicity data. This paper evaluates the use of the aquatic toxicity data submitted to the EU Registration, Evaluation, Authorisation and Restriction of Chemicals (REACH) regulation. It presents an automated computerized approach for selecting substance ecotoxicity values, building on a set of quality and reliability criteria to extract the most relevant data points for calculating the substance specific hazard values. A selected set of criteria led to the exclusion of approximately 82% of the original REACH ecotoxicological data available as of May 2015 due to incomplete initial encoding of the data by the REACH registrant, missing information such as duration of exposure, endpoint measured, species tested, and imprecise toxicity values (i.e., reported with greater than or less than signs). From an initial set of 305 068 ecotoxicity data records available in the REACH database, the final usable database contains 54 353 toxicity records (29 421 characterized as acute and 24 941 as chronic) covering 9 taxonomic groups, with algae, crustaceans, and fish representing 93% of the data. This data set is valuable for assessing the environmental toxicity of the substance contained whether through traditional substance risk assessment, product toxicity labeling, life cycle assessment (LCA) or environmental impact assessment approaches. However, the resulting loss of approximately 82% of the data suggests that changes in procedures used to generate, report, and document the data within REACH are needed to improve data utility for the various assessment approaches. The rules used to select the data to be used are the primary focus of this article. *Integr Environ Assess Manag* 2019;15:783–795. © 2019 The Authors. *Integrated Environmental Assessment and Management* published by Wiley Periodicals, Inc. on behalf of Society of Environmental Toxicology & Chemistry (SETAC).

## Key Points

- The use European Union Registration, Evaluation, Authorisation and Restriction of Chemicals regulation (REACH) ecotoxicological database has permitted to calculate unique hazard value for >6000 substances.
- The use of R programming has allowed a transparent and reproducible selection of the data (R codes are provided).
- About 83% of the ecotoxicological data registered in REACH were excluded for the final estimation of chemical hazard value.
- A significant number of chemicals (about 23%) have fewer than 2 taxonomic groups from which toxicity data are available.

## INTRODUCTION

The European Union Environmental Footprint (EU‐EF) forms a core part of the EU Commission recommendation “on the use of common methods to measure and communicate the life cycle environmental performance of products and organisations” in the context of ambitious policies stimulating sustainable production and consumption (EC 2013). It aims to ensure that environmental information in the EU market is comparable, reliable, and can be used confidently by consumers, business partners, investors, other company stakeholders, and policy makers. The EU‐EF is to be seen in the context of the Communication on Building the Single Market for Green Products and the related Recommendation (EC 2013). The EU‐EF, which is a life cycle assessment (LCA) method, addresses the environmental aspects and potential environmental impacts (e.g., use of resources and the environmental consequences of releases) throughout a product's life cycle from raw material acquisition through production, use, end‐of‐life treatment, recycling, and final disposal (i.e., cradle‐to‐grave) (ISO 2006).

In the EU‐EF, the potential toxicity impact of substances emitted during the life cycle of a product on freshwater aquatic ecosystems and on human health is assessed via the USEtox multimedia fate model (Hauschild et al. 2008; Rosenbaum et al. 2008). This model uses physicochemical and fate properties to estimate substance distributions in various environmental compartments (air, water, soil, etc.). Ecotoxicity and human toxicity (for cancer and no‐cancer) data for each substance are used with the calculated exposure to estimate potential toxicity impacts on freshwater ecosystems and humans. Recent work has discussed the need to update and expand the substance database used with the USEtox model, to improve the quality of the results and coverage of the universe of substances (Saouter et al. 2017a, 2017b). One obvious option was to use the information from substance dossiers submitted in response to the EU Registration, Evaluation, Authorisation and Restriction of Chemicals (REACH) regulation (EC 2006), as already envisaged by other authors (Askham 2012). The REACH database, available online (https://echa.europa.eu/), is an important source of physicochemical properties, partition coefficients, and half‐lives in the different environmental compartments, and of ecotoxicological and toxicological data that could be used in the USEtox model (or in any other substance toxicity models used in LCA). The advantage of using the REACH data in the context of the EU‐EF, or with any other LCA toxicity impact assessment methods, would be that registration data, which are already used for substance risk assessment, classification, and labeling of products, would be used also for environmental footprinting purposes, leading to a more consistent use of the input data gathered by EU institutions. In fact, both substance risk assessment and LCA use the same multimedia fate modeling approach to estimate potential impacts of substances in the environment. A key advantage of using the REACH database is that the procedures to generate, collect, analyze, interpret, and select high‐quality values are clearly described in European Chemicals Agency (ECHA) guidance documents and are provided to all actors that manufacture or import substances in the EU (ECHA 2015). The registration is part of a mandatory process: All substances on the EU market must be registered with the underlying principle that “no data equals no market.”

The registration process comprises searching for information, assessing its reliability and relevance, determining the classification and labeling, performing the hazard identification, thinking of additional test data, defining the exposure scenario, calculating the human and environmental risk assessment through the entire life cycle of the substance, completing the substance safety report, and communicating along the supply chain (ECHA 2016). The registration deadlines, the number of data points, and the type of data required, including short or long‐term ecotoxicity and human toxicity information, are linked to the tonnage of the substance marketed or imported into the EU, to obtain data on the highest production mass classes and highest impact relevance first.

Several authors have already used the REACH database to analyze the appropriateness of the database for LCA, to derive substance characterization factors for LCA purposes using USEtox modeling approaches (Askham 2012; Igos et al. 2014), and/or to compare substance hazard values calculated with REACH to the ones in USEtox's current organic substances database (Askham 2012; Gustavsson et al. 2017; Müller et al. 2017). They have highlighted the opportunities (a large number of substances are currently registered with physical and toxicological information) and the limitations (not all data stored in the REACH database are of high quality and test studies can be poorly documented). Actually, the REACH regulation requires that all information currently available on a substance has to be registered, including data of low quality, to ensure that every single piece of information can be taken into account when assessing the safety of a substance. However, to enable quality‐related selection of information entered into the REACH database, the regulation published extensive guidance documents to help assess the relevance, reliability, and adequacy of the information (ECHA 2015). Using REACH data for LCA and in particular the EU‐EF approach requires that the users understand the purpose of the regulation and the strategy and rules that REACH registrants need to follow to collect and assess the information.

The present paper describes the content of the REACH ecotoxicity database and the rules and selection strategy applied to generate a unique substance freshwater aquatic toxicity value (e.g., hazard value). Those values are proposed to be used for the EU‐EF and/or in any other LCA methods that address freshwater ecotoxicity impact. The comparison of potential ecotoxicity impact among products being at the heart of the objective of the EU‐EF (rather than an evaluation of compound risk), it is important that as many substances as possible that are emitted from a product have an associated toxicity value, even if this value carries a significant level of uncertainty. This requirement is to avoid having products that emit substances without toxicity values be evaluated positively (e.g., as less toxic) compared to products for which toxicity values are available for all substances. Efforts were made to find the right balance between a broad coverage of substances with toxicity values and the required minimum data quality to calculate such toxicity values. All the work to manage the database and select final toxicity values has been realized using the R studio Program (Kabacoff 2015).

## MATERIALS AND METHODS

### The REACH database

All aquatic ecotoxicity data present in the REACH database as of May 2015 were exported by ECHA from the International Uniform Substance Information Database (IUCLID 5.5; ECHA 2007) into several Excel files. The IUCLID is the key software application that both regulatory bodies and the chemical industry use for the implementation of various regulatory programs. The REACH database contained 305 068 ecotoxicity results for 7714 substances. The database includes data from acute and chronic toxicity tests performed with various taxonomic groups, derived with or without using regulatory‐adopted testing guidelines, derived from quantitative structure–activity relationship (QSAR) and read‐across methods, or obtained from the scientific literature. The extract includes all substances registered from the first 2 official registration deadlines (2010, 2013) for substances already in use at the time of the REACH enforcement. Registrations for the last deadline (June 2018) cover low‐tonnage substances for which limited test data are required. Therefore, new ecotoxicity data available from June 2018 onward are expected to be rather limited in number and relative relevance. Nevertheless, the procedure we are proposing herewith, via R programming language applied to the Excel files that the data were downloaded into, can be reapplied at any time to new extracts of the REACH database to take advantage of newly added data or dossier updates (Kabacoff 2015).

Each row of the ECHA‐exported Excel files is dedicated to the characteristics and results of a single toxicity test. Each of the 28 columns provides experimental details such as duration, reliability codes, adequacy codes, type of study, and guidelines, and corresponds to a specific data field in IUCLID 5.5. The number of available data fields in IUCLID 5.5 for each test is much higher than 28, but only the entries that were judged to be important to understand the context, quality, and results of the toxicity study for the present purposes were retained (see Supplemental Data TS1).

The export of the original ECHA database presented several practical challenges, such as information mistakenly recorded in the wrong column, missing test duration or test results, spelling mistakes in species names, lack of phylogenetic information (family, class, phylum, etc.), and durations and test results expressed in different units. The first data curation operation consisted of deleting duplicate records (42 613) and records for which duration (4418), results (8658), and species names (11 068) were absent, bringing the database to 241 633 toxicity records from 94 199 different study reports (Table 1). The list of corrections applied to the original database as well as the corresponding R codes are provided in the supplementary material (see Supplemental Data TS2 and R code).

**Table 1:** Phylogenetic composition of the REACH ecotoxicological database and number of substances, study reports, and test results per taxonomic group (as of May 2015)

Taxonomic groups	Phylum	Order	Family	Species	Substances	Study reports	Test results
Crustaceans	1	15	58	183	7387	32 594	78 654
Fish	1	22	62	212	6685	29 886	75 421
Algae	12	51	74	180	6894	20 319	59 667
Amphibians	1	2	7	34	189	588	2040
Annelids	1	12	19	30	248	1119	4106
Insects	1	7	25	52	329	1062	2624
Mollusks	1	19	35	97	447	3008	6725
Others	19	61	87	159	1244	4286	8129
Plants	4	14	17	36	399	1020	3350
Rotiferans	1	2	5	10	190	317	917
Total	42	205	389	993	7713	94 199	241 633

REACH = EU Registration, Evaluation, Authorisation and Restriction of Chemicals regulation.

Approximately 65% of the substances have been registered as “mono‐constituent,” 26% as “Unknown or Variable composition, Complex reaction products or Biological materials” (UVCB), and 9% as “multi‐constituents” (see Supplemental Data TS3). Organic and inorganic substances dominate the database with a few compounds in the “petroleum,” “organometallic,” and “element” categories. However, both the “composition” and the “type of substance” are information entered by the REACH registrants and are not always accurate. For the same substances, some registrants described the substance as monoconstituent, whereas others described it as multiconstituent or UVCB (marked as “tie” in Supplemental Data TS3). The same observation applies to the definition of “organic,” “inorganic,” “element,” “organometallic,” etc. We have applied the “majority rule” (i.e., that the nature of the material is based on the component present in the greatest amount) to propose 1 single descriptor for each substance. In case of doubt, the information added from the Organisation for Economic Co‐operation and Development (OECD) toolbox was used to assign a substance composition (OECD 2018). These entries do not change the toxicity information but only affect processes such as subgrouping of compounds, with associated changes in efforts to describe patterns in the database.

Although the total number of different biological species available in the database is 993, the most tested taxonomic groups are: crustaceans, fish, and algae (88% of the results and study report) (Table 1, see also Supplemental Data TS4 for list of species available in the REACH database, TS5 for the number of toxicity test results available per species, and TS6 for the number of taxonomic groups tested for each substance). The species group called “others” is composed mainly of bacteria and less common test species not associated with one of the taxonomic groups in Table 1. The taxonomic groups listed in Table 1 (left column) were taken from the list of suggested groups to be included in species sensitivity distribution (SSD) models standardized for use in substance safety assessment (EC‐JRC 2003). For most taxonomic groups, 3 species represent more than 60% of the available toxicity tests for that taxon (up to 83% for crustaceans; see Supplemental Data TS5).

**Figure 1:**
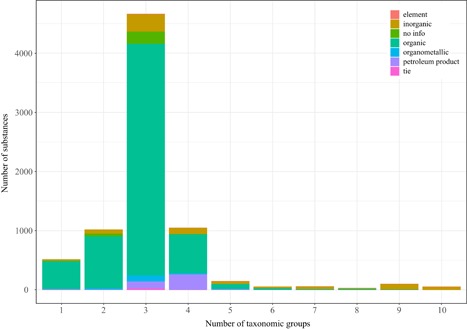
Number of substances tested plotted against the number of taxonomic groups tested per compound, discriminating the selected types of substances available in the REACH database. REACH = EU Registration, Evaluation, Authorisation and Restriction of Chemicals regulation.

For the majority of substances in the REACH database, toxicity data are available for at least 3 species (usually from fish, crustaceans, and algae), but for about 1500 substances (mainly organic substances) only 1 or 2 toxicity test data are available (Figure 1 and TS6). The distribution in Figure 1 has implications for the derivation of the SSDs (Posthuma et al. 2001; EC‐JRC 2003) underlying the derivation of the hazard value. It is recommended that there be at least 8 species from different taxonomic groups (EC‐JRC 2003).

For the majority of study reports (65%), 1 to 4 toxicity test results were recorded, usually as a result of reporting multiple endpoints (e.g., no observed effect concentration [NOEC], EC10, EC50) or durations (e.g., 48 h, 72 h, 96 h) per test. However, a significant number of study reports contained up to 20 toxicity values (from the same experiment; see TS6). This situation is usually due to the following:

- More than 1 replicate per test (the same experimental conditions have been tested more than once)
- Different experimental conditions due to variations in water hardness, pH, dissolved organic carbon (DOC), temperature, light intensity, et cetera
- Toxicity values based on nominal and measured concentrations or based on the dissolved fraction or total concentration of the substance tested (usually for metals and inorganic compounds).

### Curation of the data for the EU Environmental Footprint

Not all of the data available in the REACH database are fit for the present purpose (e.g., deriving substances hazard values to be used in EU‐EF). Depending on the goal of the work, selection rules can be applied to select appropriate endpoints and tests for the specific assessment target. The information displayed in TS1 was used to extract the endpoints considered valid to calculate final substance hazard value for use in the EU‐EF approach. Several rules have been developed and applied:

- Rule 1: Selecting high‐quality data and type of test.
- Rule 2: Selecting freshwater media.
- Rule 3: Test values presented as ranges.
- Rule 4: Selecting acute and chronic toxicity values.
- Rule 5: Using “measured” or “nominal” test concentration.
- Rule 6: Dealing with test replicates and different test conditions.

The following sections describe more in detail the rules used (see R Code in Supplemental Data).

#### *Rule 1: Selecting high*‐*quality data and type of test*

To facilitate the use of the data, REACH requests that each record should be assessed for adequacy for risk assessment, classification, labeling, etc., and for its reliability (inherent quality) using the Klimisch scoring system (Klimisch et al. 1997). For “adequacy,” test records are classified either as “key study” (46% of the REACH database), “supporting study” (30%), “weight of evidence” (14%), or “disregarded study” (3%). Seven percent of the test records were not classified for adequacy. For “reliability,” 4 levels are used to discriminate quality: k1 for “reliable without restriction” (36%), k2 for “reliable with restriction” (50%), k3 for “not reliable” (8%), and k4 for “not assignable” (2%). Three percent of the test records had no Klimisch score.

All toxicity tests described either as a key study, supporting study, and weight of evidence were retained. The test results without any adequacy descriptions were also retained if they are ranked k1 or k2 according to the Klimisch scores. Fifty‐five percent of the registered toxicity results are from experimental studies, whereas 39% are from read‐across methods and 2% are from QSAR approaches. Five percent have not been documented. All of these study types have been retained as long as the record was classified with Klimisch score k1 or k2.

#### Rule 2: Selecting freshwater media

The REACH database covers tests performed in fresh water (80%), salt water (11%), and brackish water (2%) media, with 21 569 test results (9% of the data) having no information on the exposure medium. Because the REACH regulation does not require tests to be performed in salt water, when exposure media information is missing, it was assumed that these tests were performed in fresh water (default situation). Because the purpose of our work is to provide substance toxicity values for freshwater ecosystems, only tests in or assigned to freshwater media were retained.

#### Rule 3: Test values presented as ranges

Although for the majority of test records, only 1 value was registered, for a significant number of records, test results are displayed in 2 columns with low and high value ranges without any such qualifier or with qualifier such as =, >, <, ≥, ≤, or ca. (approximately) (Table 2).

**Table 2:** Number of test values recorded with or without qualifiers for the test endpoint (effect concentration) in the REACH ecotoxicological database

Lower values		Higher values	
Qualifier	Nr results	Qualifier	Nr results
>	39 602	<	4397
≥	8068	≤	1406
ca.	3493	ca.	59
=	190 470	—	—
Total	241 633	—	5862

REACH = EU Registration, Evaluation, Authorisation and Restriction of Chemicals regulation.

A large majority of the results have a numeric value in the low range with a qualifier =, ca., ≥, or >. In contrast, only a few tests have their results expressed in the higher ranges (5862 test results). The following selections have been made to maximize the use of available data:

- When there is a lower range value with the descriptors “≥,” “ca.”, or “empty,” the lowest value is selected. If, within this group, a test has also a higher value, this higher value is ignored.
- All lower range values described as “>” are ignored (*n* = 39 602), unless the higher value is described as “≤” (*n* = 80 observations). In case of NOEC “>,” the value was kept because it still represents a concentration with no observed effect.
- All higher values described as “<” are ignored, unless the lower range value is described as “≥.” Then the lower value is used.
- When a lower range value is missing (0 or blank) and a higher value is available described as “≤,” the higher value is used.
- When a lower value is described as “≥” and the higher value is described as “≤,” the lowest value is used.
- Values expressed “<” are excluded (4397 test results).

#### Rule 4: Selecting acute and chronic toxicity values

To calculate a unique hazard value for each of the substances to be used in the EU‐EF approach, a clear separation between acute and chronic toxicity data is required to allow derivation of toxicity estimates based on acute or on chronic data only, or on both (which may be realized via application of acute to chronic extrapolation factors). In the REACH database, 2 different sections are used to report acute (short‐term) and chronic (long‐term) toxicity results for fish and aquatic invertebrate. In contrast, for algae, plants, and other aquatic organisms only 1 section is used.

There are 4 aspects to determine whether an individual toxicity test is an acute or chronic study: biological effect, endpoint, duration, and species. In principle, these 4 attributes should be combined for every study in order to assign the test data to the acute or the chronic group. Assignment to the acute or chronic groups can be difficult for some test data based solely on the data in the REACH database; however, this assignment is required to maximize the use of each toxicity test.

#### Biological effect

Biological effects are usually recorded in the REACH database in the results sections and may occur in different subsections. For algae tests, effects based on “biomass” or “growth” were partially recorded. For the large majority of toxicity test results, this type of information was not available in the REACH extract obtained from ECHA.

#### Endpoints

Ecotoxicity tests were reported using 59 different endpoints with the most frequently used being LC50, NOEC, EC50, EC10, and LOEC (Table 3).

**Table 3:** Toxicity endpoints and frequency of occurrence in the REACH ecotoxicological database covering 99% of the reported results

Endpoint	Nr results	%	Endpoint	Nr results	%
LC50	54 972	22.8%	EC90	854	0.4%
NOEC	51 951	21.5%	TTC	592	0.2%
EC50	45 621	18.9%	EL10	534	0.2%
EC10	17 302	7.2%	LL0	514	0.2%
LOEC	14 254	5.9%	ELr50	475	0.2%
EL50	9738	4.0%	EL0	442	0.2%
NOELr	5931	2.5%	ELb50	437	0.2%
LL50	5726	2.4%	ECr50	413	0.2%
EMPTY	4368	1.8%	LC20	410	0.2%
LC100	4205	1.7%	LOELr	387	0.2%
LC0	3895	1.6%	IC10	384	0.2%
EC20	3439	1.4%	IC25	348	0.1%
EC100	3238	1.3%	EL100	338	0.1%
EC0	3185	1.3%	ECb50	320	0.1%
NOEL	1967	0.8%	ChV	299	0.1%
LC10	1589	0.7%	MATC	258	0.1%
IC50	1262	0.5%	IC20	223	0.1%

b = biomass; ChV = chronic value; EC = effect concentration; EL = loading effect; EMPTY = no endpoint provided; IC = immobilization concentration; LC = lethal concentration; LL = loading rate; LOEC = lowest observed concentration; LOEL = lowest observed effect level; NOEC = no observed effect concentration; NOEL = no observed effect level; MATC = maximum acceptable toxic concentration; r = growth rate; REACH = EU Registration, Evaluation, Authorisation and Restriction of Chemicals regulation; TTC = toxicity threshold of concern.

When more than 1 endpoint was reported for the same test, the following rules were used:

- For acute and chronic median effect (50% effect) tests, endpoints usually reported are effect concentration (EC50), immobilization concentration (IC50), and lethal concentration (LC50). If a single test reports all 3 endpoints (rare situation), the order of preference was EC50 > LC50 > IC50. This prioritization is somehow arbitrary and was done to facilitate the processing of tens of thousands of data records. If, for the same substance or the same species, 1 test reports an EC50 and a second test reports an IC50 (or LC50), both endpoints were included in the calculation of a species geometric mean test value. For algae, the 50% effect can be based on growth rate (ECr50) or biomass (ECb50). If both values are reported, growth rate was selected because this is done in environmental risk assessment.
- For chronic tests, endpoints are more diverse and priority was given as follows: ECr10 > ECb10 > EC10 to EC20 > NOEC > LOEC > maximum acceptable toxic concentration (MATC) > chronic value (ChV) > toxicity threshold of concern (TTC). The EC*x* are derived from an effect–concentration relationship and are more precise than NOEC or LOEC, which correspond to tested concentrations. For this reason EC*x* are always used in priority. If, for the same substance or the same species, one test reports an LC10 and the second test reports NOEC (or any other chronic endpoint), both endpoints were used to calculate a species geometric mean test value.
- This pooling of endpoints is consistent with the approach proposed by Posthuma et al. (2019).

#### Duration

**Figure 2:**
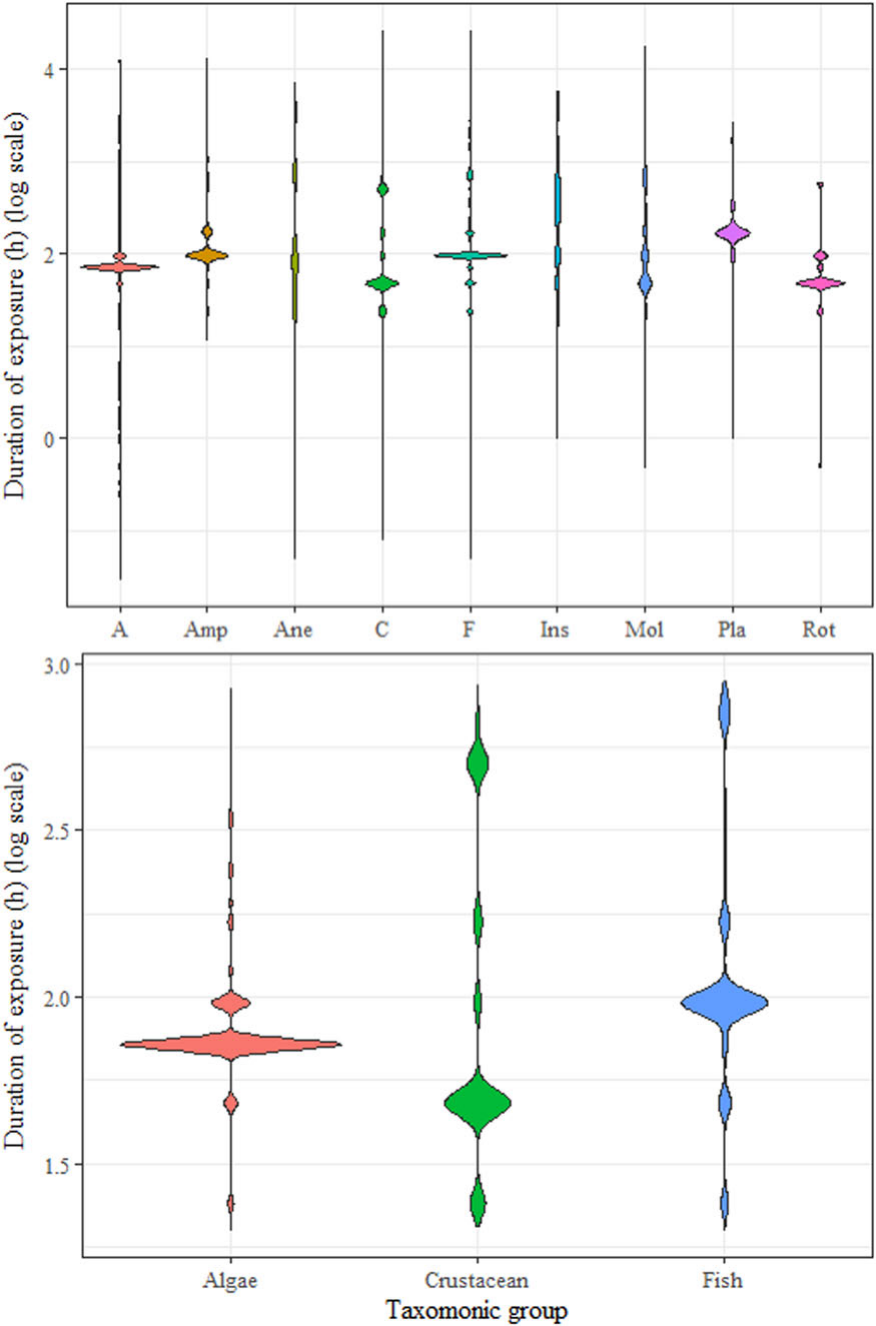
Available duration of toxicity test exposures in hours for each taxonomic group available in the REACH database. The violin plot shows the full distribution of the test duration data for the different groups. The size of diamond shapes for each species indicate the relative number of data reported for a specific duration. The vertical lines represent the range of the data. A = algae; Amp = amphibians; Ane = anellids; C = crustaceans; F = fish; Ins = insects; Mol = mollusks; Pla = plants; REACH = EU Registration, Evaluation, Authorisation and Restriction of Chemicals regulation; Rot = rotifers.

Within each taxonomic group, the reported duration of exposure varies from minutes to months for tests registered in the “short‐term” and “long‐term” IUCLID section (Figure 2, top). In the IUCLID database, users are invited to register their data either in the short‐term or in the long‐term section. There are, however, inconsistencies within the database in decisions about whether individual toxicity tests were short (i.e., acute) or long term (chronic) in duration. To provide consistency, REACH designations of short and long term were ignored and rules were established to assign tests based on duration, endpoint, biological effect, and species. For fish, algae, and crustaceans, the most frequent duration corresponds to 96 h for fish acute, 28 d for fish chronic, 48 h for crustacean acute, 21 d for crustacean chronic, 72 h for algae acute (as EC50), 72 h for algae chronic (as NOEC or EC*x*) (Figure 2, bottom). For the algae, it could be argued that the EC50 determined at 72 h is a chronic endpoint (algal cells divide many times in 72 h), although from a regulatory point of view they are considered acute and NOEC or EC10 are considered chronic endpoints (EC‐JRC 2003). The use of duration limits to separate acute from chronic is consistent with a recent attempt to use REACH data to calculate USEtox substance hazard values (Müller et al. 2017).

The standard recommended test durations are based on OECD, American Society for Testing and Materials (ASTM), United States Environmental Protection Agency (USEPA), and other standard aquatic toxicity test methods that use specific durations to assign acute and chronic exposures.

For each taxonomic group, a specific range of exposure (together with endpoint and biological effect) was used to pool the toxicity data into acute and chronic exposure categories (Table 4). Reported exposure durations typically match the official standard recommended durations, allowing for some variation due to the need to stop or prolong a test because of test observations and practical concerns (e.g., staffing). The use of a duration range to assign acute or chronic ensures that no data are excluded because the duration may vary from the standard. For fish, invertebrates, and algae, those values align with standard test guidelines. For the other taxonomic groups, the selection of the acute and chronic durations was based on the life cycle stage of each taxonomic group (based on the dominant species). Assignment of rotifer toxicity data into acute or chronic was based solely on endpoints.

**Table 4:** Proposed endpoint and duration ranges to distinguish between acute and chronic exposure per taxonomic groups

Taxonomy groups	Acute endpoint and duration	Chronic endpoint and duration
	Endpoint EC50eq: EC50, LC50, IC50	Endpoint Chronic EC50eq: EC50, LC50, IC50 Endpoint Chronic NOECeq: EC10 to EC25, LC5 to LC25, NOEC, LOEC, MATC, TTC, ChV
Algae	≥40 h and ≤120 h	≥40 h and ≤120 h
Crustaceans (mainly *Daphnia*)	≥40 h and ≤120 h	≥168 h
Fish	≥40 h and ≤120 h	≥168 h
Mollusks	≥24 h and ≤96 h	>96 h
Amphibians	≥24 h and ≤96 h	>96 h
Annelids	≥24 h and ≤96 h	>96 h
Insects	≥24 h and ≤96 h	>96 h
Plants	≥48 h and <120 h	>120 h
Rotifers	≥40 h	≥40 h

ChV = chronic value; EC = effect concentration; IC = immobilization concentration; LC = lethal concentration; LOEC = lowest observed concentration; NOEC = no observed effect concentration; MATC = maximum acceptable toxic concentration; TTC = toxicity threshold of concern.

#### Rule 5: Using “measured” or “nominal” test concentration

For the majority of the test results (44%), the toxic effect concentration was expressed as nominal (i.e., the target concentration at the start of the test), whereas 39% of the test results were reported as measured concentration (analytically verified test substance concentrations) (Table 5).

**Table 5:** Number of test results expressed as a specific type of substance measurement in the test media for the REACH ecotoxicological database

Concentration as	Nr test results	%
Nominal	106 513	44%
Measured (arithmetic mean)	43 250	18%
Measured (not specified)	30 908	13%
Empty	28 514	12%
Measured (geometric mean)	10 652	4%
Measured (initial)	8317	3%
No data	5568	2%
Estimated	4831	2%
Measured (twa)	2886	1%
Acid equivalent	194	0%

REACH = EU Registration, Evaluation, Authorisation and Restriction of Chemicals regulation; twa = time‐weighted average concentrations.

In the context of a substance safety assessment, it is critical to base the effect value on the most relevant tested concentration (i.e., measured) because some compounds can be degraded and/or biodegraded volatilized, or adsorbed to test vessels. When toxicity data based on measured concentrations are not available, results from nominal concentrations provide the next best data for use in the assessment. Similarly, for the purpose of the EU‐EF, where products are compared to each other, it is important to have data on as many substances with toxicity data as possible. Retaining only measured concentration would eliminate 44% of the data in the database. Therefore, we pooled nominal and measured concentration. However, if in the same test, toxicity values are reported for both nominal and measured concentrations, measured values are selected as priority. For some substances, such as metals, it is essential to base the toxicity assessment on the fraction of metals dissolved in the water media. In this case, if for the same test, values are reported for both total and dissolved fractions, the latter is systematically retained.

#### Rule 6: Dealing with test replicates and different test conditions

The information regarding test replicates and test conditions (i.e., testing different temperature, different pH, different DOC) is recorded in a “free text” field without a predefined structure that could allow automated selection and treatment of different experimental test conditions. For the calculation of the substance toxicity value, we opted for arithmetic means of all tested conditions (average of replicates, average toxicity for all water hardness tested, average toxicity for all DOC tested, etc.) because different test conditions normally represent to some extent the diversity of situations in the real environment. Taking an average is a way to acknowledge that the diversity of real environmental conditions can be represented by the average data point.

## RESULTS AND DISCUSSION

**Figure 3:**
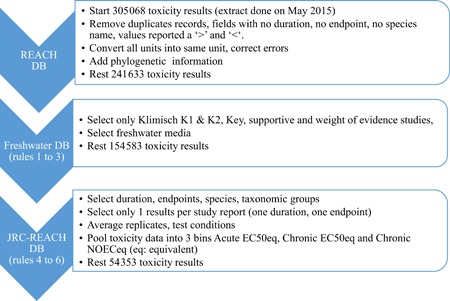
Three steps applied to the initial REACH database to build the final JRC‐REACH database from which substance‐specific hazard value could be derived. DB = database; JRC = Joint Research Centre; REACH = European Union Registration, Evaluation, Authorisation and Restriction of Chemicals regulation.

The selection procedure to build the final ecotoxicity database is summarized in Figure 3. Using the rules described previously, 2 successive database versions were created from the initial REACH database. The first one (Freshwater database) contained only high‐quality data performed on freshwater species (Rules 1 to 3) with 154 583 toxicity test records (about 50% of the initial REACH database). Because these modifications are coded in R, the selection procedure applied on the REACH database can be easily modified to address different needs. For example, many test results have been excluded because the reported value was expressed as “higher than” or “lower than.” In a substance safety assessment, these values cannot be used to precisely define an equivocal substance safety conclusion. However, in the context of the EU‐EF, these values may still provide interesting comparative information: If a substance has a toxicity value >100 mg/L, it could be concluded that it is not toxic to aquatic biota compared to those that have a value of 0.1 mg/L.

A second database (the JRC‐REACH database, using Rules 4 to 6) contains the acute and chronic data that are considered of appropriate quality to be used to derive a hazard value for each of the substances. Toxicity data were then pooled into 3 categories: acute EC50equivalent (EC50_eq_; 29 412 test results), chronic EC50_eq_ (3197 test results), and chronic NOEC_eq_ (21 744 test results) (Table 6). This subset of selected data represents approximately 17% of the initial number of tests in the REACH database.

**Table 6:** Total number acute EC50_eq_, chronic EC50_eq_, and chronic NOEC_eq_ toxicity in the final ecotoxicity database^a^

	Acute EC50_eq_		Chronic EC50_eq_		Chronic NOEC_eq_	
Taxonomic group	Nr substances	Nr test results	Nr substances	Nr test results	Nr substances	Nr test results
Algae	3548	6528	na	na	4998	9772
Amphibians	52	213	33	148	43	209
Annelids	98	259	6	6	50	67
Crustaceans	3590	11 098	886	1680	2468	5718
Fish	3153	10 556	419	949	1366	4432
Insects	171	372	15	20	179	356
Mollusks	147	327	63	120	183	455
Plants	37	39	137	235	260	525
Rotifers	14	20	29	39	131	210
Total	—	29 412	—	3197	—	21 744

eq = equivalent; NOEC = no observed effect concentration.

^a^ Total unique substances = 6461.

**Figure 4:**
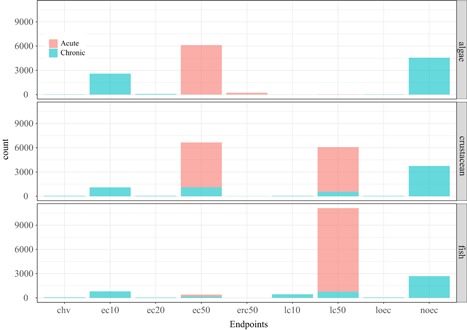
Main endpoints reported for the fish, crustaceans, and algae in the JRC‐REACH database. JRC = Joint Research Centre; REACH = European Union Registration, Evaluation, Authorisation and Restriction of Chemicals regulation.

Others have built similar ecotoxicity databases aimed at comparing existing USEtox hazard values (HC50) with the ones calculated using the REACH database (Müller et al. 2017). Although the selection procedure was similar to the one applied here, the Müller study was restricted to those substances present in the current USEtox and REACH databases (i.e., ). In contrast, we have applied the selection procedure to the whole REACH database (7713 substances) with the aim of eventually calculating new hazard values using the USEtox approach (or any other life cycle impact assessment [LCIA] model) for as many substances as possible. The work from Müller et al. used only EC50, IC50, and LC50 to build acute and chronic data bins, whereas we are proposing to extend the number of endpoints to make use of as much as possible of the toxicity data generated and registered under REACH. Another important addition to the previous work is the creation of a new data bin using all existing chronic endpoints such as NOEC, LOEC, and EC*x*. Those endpoints represent 40% of the substance toxicity database (Table 6 and Figure 4) and are considered to represent an appropriate endpoint for deriving substance hazard value based on the preferred chronic exposure data. The approach used by USEtox to retain only chronic EC50 (relatively rare data) and extrapolate acute EC50 to chronic EC50 via an extrapolation factor of 2 for all organic substances may have lower reliability, due to the lower numbers of data available, than the currently proposed approach for LCA (Müller et al. 2017).

**Figure 5:**
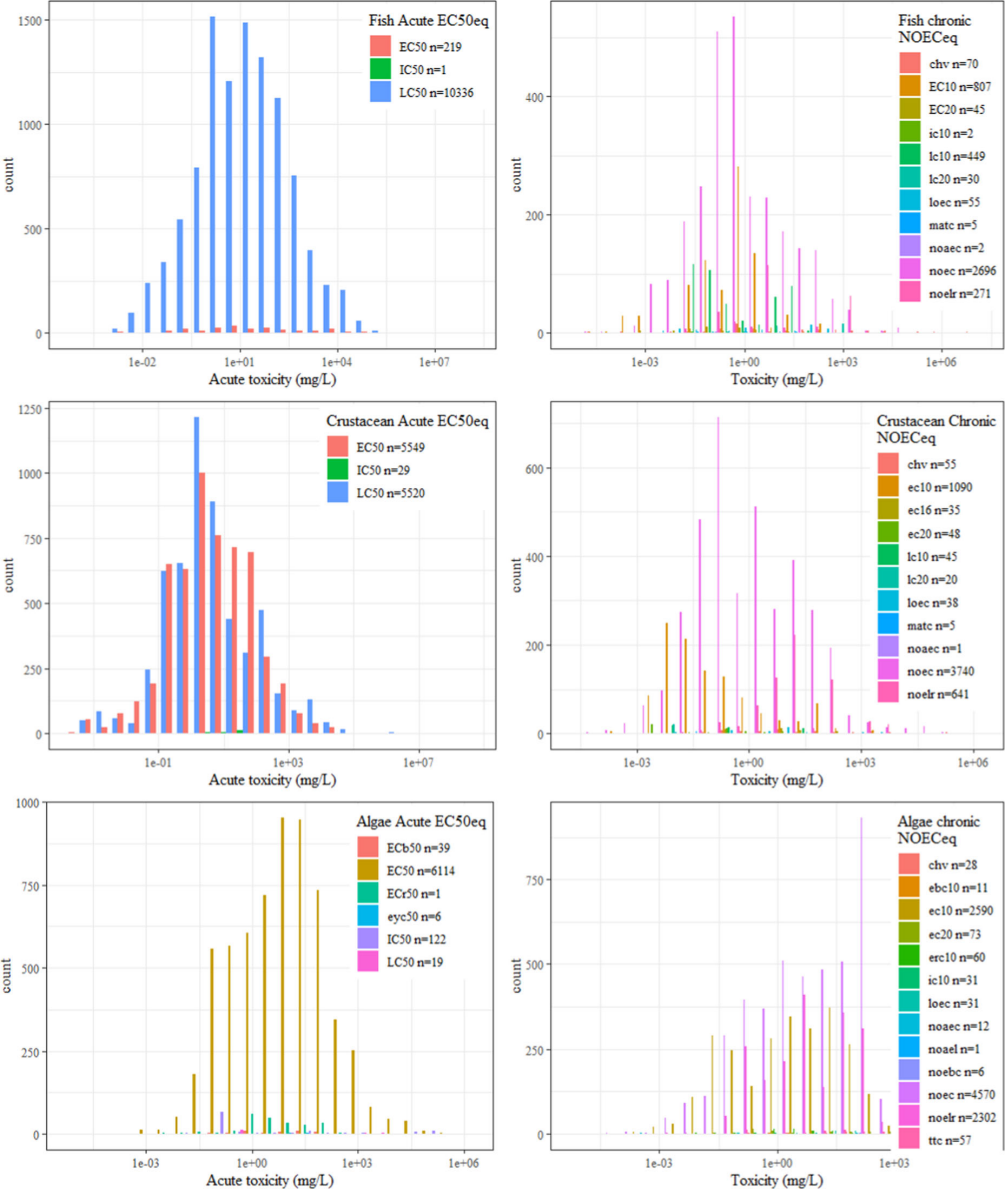
Distributions and number of selected toxicity endpoint values for acute and chronic exposure for the main 3 taxonomic groups (fish, crustaceans, and algae) for all the substances in the JRC‐REACH database. ChV = chronic value; IC = immobilization concentration; JRC = Joint Research Centre; MATC = maximum acceptable toxic concentration; NOAEC = no observed adverse effect concentration; NOEC = no observed effect concentration; NOELr = no observed effect level based on growth; REACH = European Union Registration, Evaluation, Authorisation and Restriction of Chemicals regulation.

For the 3 most tested taxa, the availability of endpoints for acute and chronic exposure are presented in Figure 5. For fish acute EC50_eq_, the dominant endpoint in the final database is LC50 (96%) with the remaining tests reporting EC50 values. For crustaceans, the dominant endpoint is EC50 (78%) with the remaining data points being LC50 (20%) and IC50 (2%). For algae, more endpoints are available, mainly because EC50s can be reported based on biomass (ECb50) or growth rate (ECr50), but the dominant endpoint is EC50 (96%). The overlap between all retained endpoints to describe acute toxicity suggests that building a substance‐specific database aggregating slightly different endpoints (regarding their name, not their interpretation) should be acceptable.

For the fish chronic NOECeq, 91% of the test results are expressed as a NOEC. Few test results are expressed as LOEC (2.5%), ChV (4.3%), or EC10 (1.4%). Surprisingly, the EC10 value appeared to be sometimes lower than the NOEC for compounds tested multiple times on a species (Figure 5). Further, the distribution of the LOECs overlaps the NOECs. For crustaceans, 88% of the data points are NOECs, whereas 9.4% are EC10 values. The rest of the endpoints are EC16 and 20, LOEC, and ChV. For chronic algae, 52% of the data points are reported as NOEC and 45% as EC10 values. The NOEC and EC10 overlap, suggesting these 2 endpoints as equivalent. With the comparisons of endpoints within a taxa and exposure duration (acute, chronic), it is important to keep in mind that data points are obtained on a diversity of substances and species.

When performing a substance safety assessment, all the chronic endpoints that were included in the chronic NOEC_eq_ bins are not commonly considered equivalent. However, on the basis of numerical inspections it is becoming more and more evident that NOEC and EC10 to EC20 are estimates of the same type of sensitivity or effect information (Azimonti et al. 2015; Beasley et al. 2015). The LOEC usually is not considered as equivalent to NOEC because the LOEC corresponds to the next higher test concentration where a statistically significant effect occurs. However, based on the fact that LOEC is not so different from the rest of the endpoints (see Figure 5), we are proposing to include this endpoint in the defined chronic NOEC_eq_ bin when no NOEC is available. The numerical impact of this choice on the hazard value should be minimal given that the number of LOEC is rather limited (9% of the chronic endpoint).

An example of the data selection procedure is given in Supplemental Data for the substance formaldehyde (Chemical Abstracts Service [CAS]: 50‐00‐0) to illustrate how data are finally selected. In the initial REACH database, 94 toxicity results were available for this substance (Supplemental Data TS7). After the first selection rules (Rules 1 to 3), 51 tests remained for the present purpose (Supplemental Data TS8). Twenty‐one test records were eliminated because no endpoint was reported, although 14 of those were classified as Klimisch k2 studies. Two more tests were eliminated because the results was reported as “greater than.” Thirty‐four test endpoints were excluded because the tests were classified as Klimisch k3 and k4. Finally, 9 tests were excluded due to the use of salt water as the test medium. The selection of the final acute and chronic data resulted in a final JRC‐REACH database of 15 test results (12 acute, 1 chronic EC50, and 2 chronic NOEC) (Supplemental Data TS9). Twelve of these test results are derived from 1 single value per test, whereas for 3 tests, the values are based on average on 2 or 3 replicates.

## CONCLUSIONS

The EU's REACH provides of the largest ecotoxicity database currently available to perform substance safety assessments and hazard classifications. However, not all of the data are fit for these and other uses, including the derivation of hazard values for LCA and the EU‐EF. A substantial data curation and interpretation approach was designed to prepare and subselect the data that are most useful for the purpose of LCA and the EU‐EF (derivation of substance hazard values). A theoretically motivated selection procedure was shown to result in the exclusion of approximately 83% of the original data. The high‐quality remaining data are retained for the final calculation of hazard values. Given that the loss of data implies substantial tradeoffs regarding the number of compounds for which a hazard value can be derived, we programmed the selections rules, coded in R, so that the criteria can be easily modified. The existing code can easily be reapplied on a new extract of the IUCLID database as additional or updated data become available.

The following conclusions could be drawn:

- To our knowledge, this is the first attempt to make use of all REACH registered data (>300 000 test results) to derive ecotoxicity hazard values for thousands of substances by using a fully transparent and reproducible selection procedure, thanks to R programming (available in Supplemental Data). This code can be easily reapplied or modified to accommodate different needs.
- The procedure for registering toxicity data into the IUCLID system should be improved to allow an automatic and computerized extraction of the database. Currently many fields are missing data or contain inaccurate information, sometimes preventing the inclusion and use of high‐quality data (classified as k1 or k2). Access to the original dossier could eventually allow retrieval of missing information, but such extra steps are time consuming and should be avoided in good‐quality database design and management.
- For the majority of substances, typically only 3 trophic levels have been tested (fish, crustaceans, and algae) with the exception of some inorganics and metals for which more than 10 different taxonomic groups are available. It is usually recommended to derive SSDs with a relatively higher number of taxonomic groups (*n* = 8; EC‐JRC 2003). Therefore, the use of the SSD approach to derive substance HC50 values as recommended by USEtox would carry a high level of uncertainty for those substances.
- For a significant number of substances, only 1 or 2 toxicity data points are available. This is fewer than the number commonly required for regulatory substance safety assessment. Deriving a chemical toxicity value from few data points carries high uncertainty and makes toxicity comparison between chemicals less reliable. However, toxicity estimation for those substances was still performed in order to avoid that in the EU‐EF products emitting a significant share of substances without toxicity values are evaluated positively compared to products for which toxicity values are available for all substances. Efforts have been made to find the right balance between a broad coverage of substances with toxicity values and the required minimum data quality to calculate such toxicity values.
- The database created using the selection procedure allows retrieval of all potentially high‐quality toxicity value for 6461 substances, although 83% of the initial database has been omitted. Further work needs to be conducted to demonstrate the robustness of the selection procedure by comparing the hazard value calculated with this database and with other existing databases, such as USEtox or the data used to perform environmental risk assessment or classification according to the Classification, Labelling and Packaging (CLP) regulation or to the Global Harmonization System (EC 2008; UN 2017). Assuming that there is a true but unknown distribution of sensitivities, such robustness assessments would boil down to evaluating convergence of different data collation and curation methods toward a constant value.

The database resulting from the application of the selection procedure has been used 1) to calculate new acute‐to‐chronic ratios related to different modes of action of the substances and 2) to compare different approaches to derive substance toxicity hazard values and to compare those with current CLP ranking, and finally to calculate ecotoxicity characterization factors for the EU‐EF (Saouter et al. 2018, 2019).

## Disclaimer

The authors declare no conflicts of interest.

## Data Accessibility

All ecotoxicity data have been provided by the European Chemical Agency (ECHA) are available on the ECHA dissemination website (https://echa.europa.eu/fr/home).

## SUPPLEMENTAL DATA

**TS1.** List of the fields from IUCLID provided by ECHA with each test result.

**TS2.** Initial corrections applied to the original REACH ecotoxicological database.

**R Code.** Codes for creating the freshwater ecotoxicity database.

**TS3.** Total number and type of substances in the REACH ecotoxicological database (7713 substances).

**TS4.** List of species available in the REACH database.

**TS5.** Number of test results per species.

**TS6.** Number of available taxonomy groups per substances.

**TS7.** Ecotox data available in REACH for CAS: 50‐00‐0; EC: 200‐001‐8, Formaldehyde.

**TS8.** Ecotox data retained for CAS 50‐00‐0 after first selection.

**TS9.** Ecotox data retained for CAS 50‐00‐0 after final selection.

## Supporting information

This article contains online‐only Supplemental Data.

This article contains online‐only Supplemental Data.
